# Application of artificial intelligence in eosinophilic esophagitis

**DOI:** 10.3389/fimmu.2025.1712113

**Published:** 2025-11-10

**Authors:** Xiaoming Xu, Hualei Chen, Yun Chen, Lijuan Fan

**Affiliations:** 1 Department of Gastroenterology, Jining No. 1 People’s Hospital, Jining, China; 2 Department of Gastroenterology, Affiliated Hospital of Jining Medical University, Jining Medical University, Jining, Shandong, China

**Keywords:** artificial intelligence, diagnostic, eosinophilic esophagitis, endoscopy, review

## Abstract

Artificial intelligence (AI) shows great promise in eosinophilic esophagitis (EoE) management. It enhances diagnostic accuracy and consistency in endoscopic and histopathological analyses, with performance comparable to or exceeding non-experts. AI aids in standardizing assessments like EREFS and EoEHSS, identifies molecular phenotypes and novel biomarkers, and predicts treatment responses, facilitating precision medicine. However, challenges exist: “black box” issues demand explainable AI (XAI) for trust; validation in large, diverse cohorts, ensuring model generalization, and regulatory approval are crucial; data governance, privacy, and algorithmic integrity require attention. Future priorities include researching pediatric populations, improving treatment response prediction, and developing non-invasive monitoring tools. An integrated multimodal AI platform may transform EoE care from reactive to proactive, personalized approaches.

## 1 Introduction

Eosinophilic Esophagitis (EoE) is a chronic inflammatory disorder of the esophagus driven by an immune response to specific antigens. Its characteristic pathological feature is a substantial infiltration of eosinophils within the esophageal mucosal tissue (1). In recent years, EoE’s global incidence has surged, now rivaling inflammatory bowel disease in Western nations (2). The pathophysiological mechanisms of EoE are complex, primarily driven by a combination of genetic susceptibility, environmental triggers, and impaired esophageal barrier function (3, 4). Adults with EoE typically present with dysphagia and food impaction, whereas children exhibit more diverse symptoms such as vomiting, abdominal pain, and feeding difficulties, all significantly impairing quality of life (2). Early diagnosis and timely individualized intervention for EoE are becoming increasingly important in clinical practice.

Although the Endoscopic Reference Score (EREFS) is a valuable tool, a normal endoscopy does not exclude EoE. Therefore, the definitive diagnosis always depends on histologic assessment of multiple esophageal biopsies (5). Currently, diagnosing and managing EoE remains clinically challenging in practice. First, diagnostic delay is a major issue in EoE. This is largely due to non-specific symptoms and patients’ self-management, which can mask the severity of the condition (6). Second, the need for repeated invasive endoscopies with biopsies adds significant strain on patients and healthcare systems alike (7). Finally, the assessment of EoE is subjective and variable (8). Subtle features such as esophageal rings, longitudinal furrows, white exudates, edema, and strictures can be easily overlooked by less-experienced endoscopists, leading to significant inter-observer variation (9). Furthermore, as eosinophils in EoE are often focally distributed, relying solely on peak counts per high-power field may fail to accurately reflect disease severity (10). These factors delay EoE diagnosis and treatment, and prolonged inflammation raises the risk of irreversible complications like fibrosis and strictures.

Artificial intelligence (AI), a branch of computer science, aims to simulate human cognitive functions such as learning and problem-solving (11). Machine learning serves as a key component, enhancing system performance through data-driven methods (12). Deep learning (DL), a subset of machine learning, uses multi-layer artificial neural networks (ANNs) such as convolutional neural networks (CNNs), which excel in image processing tasks like medical image analysis, to learn hierarchical features from data (13, 14). AI is transforming medicine, with notable impact in gastroenterology (15). It improves endoscopic diagnosis, increases lesion detection, and advances precision medicine (16, 17). AI offers multiple advantages in the management of EoE. It can enhance diagnostic accuracy and consistency by analyzing endoscopic and histopathological images (14), standardize evaluations through automated calculation of metrics such as the EREFS score and EoEHSS grade (18), and integrate multimodal data, including clinical, endoscopic, histological, and molecular information, to identify disease subtypes and characterize heterogeneity (19). Furthermore, it shows potential in predicting responses to specific treatments such as dietary or pharmacological interventions, thereby supporting personalized therapeutic decisions (20). Additional prospects include enabling non-invasive monitoring and improving research efficiency (21). AI integration enhances diagnostic accuracy and clinical efficiency to improve long-term patient outcomes.

## 2 AI-assisted endoscopic diagnosis of EoE

### 2.1 Optimizing EoE detection: development and validation of deep learning models

In endoscopic diagnosis of EoE, computer-aided diagnosis (CAD) systems can automatically detect subtle features like edema and annular rings that may be missed visually. By serving as a “second observer,” these systems improve lesion detection and help minimize diagnostic oversight (9, 22). In recent years, convolutional neural networks (CNNs) have been increasingly applied to EoE diagnosis due to their strong image analysis capabilities. By automatically learning hierarchical features, from edges and textures to complex shapes, CNNs can detect subtle endoscopic signs of EoE that may be overlooked visually (10). Models such as ResNet, DenseNet, and U-Net have been trained on annotated endoscopic images labeled with EoE status and EREFS criteria to support this task (23). Okimoto et al. developed a CAD system using ResNet50, trained on 1192 EoE and 1192 normal esophageal images. On an independent test set, the model demonstrated 94.7% image-based accuracy (90.8% sensitivity, 96.6% specificity), along with case-based sensitivity and specificity of 94.9% and 99.0%, respectively. Clinically, these results are highly relevant: the high sensitivity indicates a strong ability to identify EoE cases, reducing the likelihood of missed diagnoses, while the exceptional specificity helps avoid unnecessary biopsies in non-EoE patients (17). In Guimarães et al.’s study, a CNN model was trained to distinguish between three categories of images: normal esophagus, active EoE, and esophageal candidiasis. The model achieved an overall accuracy of 91.5% and an area under the curve (AUC) of 0.966, outperforming the participating human endoscopists. This model covers the full range of common differential diagnoses encountered in clinical practice, thereby expanding the diagnostic scope of the model (17). In Römmele et al.’s study, a deep learning algorithm named AI-EoE was developed for binary classification (EoE vs. normal). On an external validation dataset, the model achieved a sensitivity, specificity, and accuracy of 0.93 each, with an AUC of 0.986 (9). By employing a multicenter validation approach, this study enhanced the model’s generalization capability. Daniel et al. employed deep learning to model and validate 1,066 whole-slide images from 400 patients across multiple institutions. The model demonstrated excellent performance and successfully addressed two major challenges in EoE diagnosis and digital pathology: the need to simultaneously detect multiple small features and the capability to efficiently analyze entire slides (24).

### 2.2 Enhancing diagnostic capabilities: integrating EREFS scoring into AI

The Eosinophilic Esophagitis Endoscopic Reference Score (EREFS) is a validated clinical tool for grading the severity of endoscopic inflammation (9) and has been shown to correlate with histological severity and treatment response (25). While EREFS has improved the standardization and diagnostic utility of endoscopic assessments, it cannot replace histological biopsy for confirming EoE. This is because the inter- and intra-observer agreement for scoring individual features only reaches moderate to good levels, indicating non-negligible variability in the scoring process (5). Artificial intelligence technology helps address the limitations of the EREF scoring system in practical applications. Römmele et al. went beyond simple binary classification, developing the AI-EoE-EREFS model. During training, this model incorporated specific auxiliary branches for each EREFS feature (e.g., severity of edema, rings, exudates, furrows, and strictures) (9). This integration significantly enhanced the model’s performance. Studies showed that in external validation, AI-EoE-EREFS achieved a sensitivity of 0.96, specificity of 0.94, accuracy of 0.95, and an AUC as high as 0.992 (12). These results confirm that integrating EREFS with AI enhances both the diagnosis and severity assessment of EoE.

### 2.3 Performance comparison: AI vs. human endoscopists

Current studies indicate that AI models matches the performance of experienced endoscopists in identifying EoE and exceeds that of less-trained practitioners (26). In Römmele et al.’s study, both AI-EoE and AI-EoE-EREFS performed significantly better than novice endoscopists and senior specialists on the same set of images (9). Similarly, the accuracy of Guimarães et al.’s model (91.5%) was higher than that of the endoscopists they tested (83.1%) (17). These findings highlight the significant role of AI in standardizing diagnostic quality and suggest that it can serve as a valuable training and auxiliary tool for endoscopists with varying levels of experience (27) (**Table 1**).

**Table 1:** Application of artificial intelligence in eosinophilic esophagitis.

No	Author	Year	AI-built models	Aim
AI-assisted endoscopic diagnosis of EoE				
1 (26)	Guimarãe	2022	CNN	Trained and tested a Convolutional neuronal network (CNN) using real-world endoscopic images to diagnosis EoE
2 (9)	Römmele	2022	A deep learning-based AI model	Develop and validate a deep learning-based AI model for EoE detection, EREFS quantification, and comparison with endoscopists’ performance.
3 (27)	Okimoto	2022	CNN based on ResNet50	Constructed a computer-assisted diagnosis (CAD) system using a convolutional neural network (CNN) and evaluated its performance to diagnosis EoE.
4 (24)	Daniel	2022	Deep learning	To develop and validate a machine learning model for EoE identification, quantitation and diagnosis.
EoE histopathological analysis based on AI technology				
1 (18)	Archila	2023	AI based digital pathology model	Evaluating histologic features related to EoE
2 (32)	Zhang	2024	MC-AI (Machine Learning)	Identifying EoE mast cells, analyzing their distribution in esophageal biopsies.
3 (33)	Wu	2024	Machine Learning (XGBoost)	Evaluating pivotal molecular markers that may facilitate the diagnosis of EoE
4 (30)	Larey	2022	AI platform	Predict EoE activity and classify pathological severity
5 (10)	Xiong	2024	Open-EoE (Ensemble of CNNs)	An efficient and accurate open-source toolkit for peak eosinophil counts has been developed
6 (14)	Czyzewski	2021	Deep CNN	Biopsy classification based on global characteristics
7 (29)	Adorno	2021	UNet++(CNN)	Analysis of spatial biomarkers
Application of AI in EoE prognosis and precision medicine				
1 (20)	Visaggi	2024	machine learning	Early diagnosis of EoE
2 (40)	Halder	2022	machine learning	Prediction of treatment response and prognosis
3 (34)	Wang	2025	machine learning	Early screening for EoE
4 (36)	Sallis	2018	Machine Learning (Random Forest)	Diagnosis and classification
5 (37)	Sallis	2018	Machine Learning	Molecular typing and prognosis prediction
6 (35)	Shoda	2018	Machine Learning	Internal molecular phenotyping

## 3 AI-based histopathological analysis of eosinophilic esophagitis

### 3.1 Deep learning-based automatic quantification of eosinophils (PEC)

Esophageal biopsy is central to EoE diagnosis and monitoring, yet manual eosinophil counting remains limited by its time-consuming procedures, significant inter-observer variability, difficulty in identifying peak regions, and inconsistent microscopic field sizes (18, 27). The current gold standard for diagnosing EoE is manual counting of peak eosinophils under a microscope, with a diagnosis confirmed when the count reaches or exceeds 15 per high-power field (hpf) (28). However, this diagnostic process is cumbersome and subject to subjective variability. The U-Net architecture is particularly effective for this application due to its strength in pixel-level localization. This capability enables precise delineation of individual eosinophil boundaries, which is essential for addressing challenges such as cell clustering and differentiating eosinophils from other cell types in densely packed tissue regions (10). The Open-EoE toolkit utilizes an ensemble learning strategy integrating multiple object detection models including Faster R-CNN, Mask R-CNN, and CenterNet, enabling efficient and accurate provision of peak eosinophil counts from WSIs and addressing the limitations of manual counting (10). A study by Reed et al. proposed a U-Net-based system, which was trained on hematoxylin-eosin (H&E)-stained biopsy images with annotated eosinophil locations to learn the identification and segmentation of individual eosinophils. In subsequent processing, we counted eosinophils within the predefined HPF field to estimate the peak eosinophil count (PEC) (21). The system automates eosinophil counting and produces statistical data to measure disease severity and progression, highlighting the value of objective, automated histological analysis (29). Studies show that AI models count eosinophils with accuracy comparable to pathologists, reliably identifying samples that meet EoE diagnostic criteria. This method reduces the time and subjectivity of manual counting while providing heatmaps to visualize cell distribution (17).

### 3.2 Application of AI in comprehensive analysis of EoEHSS features

The role of AI in EoE histopathological analysis is not limited to PEC quantification. To enable a more comprehensive evaluation of histological changes in EoE, the research team also developed and validated the EoE Histological Scoring System (EoEHSS) (30). EoEHSS is a semi-quantitative scoring system that assesses multiple histological features beyond PEC, including eosinophilic abscesses, surface epithelial alterations, basal zone hyperplasia, spongiosis, lamina propria fibrosis, and degranulation (18). Nevertheless, its clinical adoption remains challenging due to the complexity of the scoring protocol, the number of parameters required, and the time needed for assessment. However, with the advent of AI technology, segmentation and classification models based on deep learning are well-suited for automatically detecting and quantifying all these features on whole-slide images (WSIs). This approach overcomes the limitations of manual PEC counting and manual EoEHSS scoring, providing a richer and more standardized histological assessment than manual PEC counting (21). Archila et al. developed an AI-based digital pathology model to evaluate histological features of EoE. By integrating object detection, semantic segmentation, and instance segmentation, the model systematically analyzes tissue cellular composition, epithelial and lamina propria changes, as well as eosinophil activity. It demonstrates strong PEC and quantitative assessment of multiple EoEHSS histological features, achieving a level of accuracy comparable to experienced gastrointestinal pathologists (31).

### 3.3 AI-based analysis of global features and other immune cells

EoE pathology encompasses not only eosinophil infiltration but also features such as basal cell hyperplasia, dilated intercellular spaces, and lamina propria fibrosis, the accurate quantification of which remains difficult with traditional methods (3). AI offers a novel approach to this problem. Models based on deep convolutional neural networks (DCNNs) can accurately classify EoE biopsy specimens not only by detecting localized eosinophils but also by learning the “overall histological features” of the entire biopsy slide (14, 21, 30). A study utilized an AI platform to achieve semantic segmentation of eosinophil and basal cell regions within tissue sections, subsequently developing novel spatial biomarkers (such as eosinophil spatial density, basal cell peak area, and distribution patterns). These markers outperformed traditional PEC metrics, with the constructed model achieving 86.7% accuracy in histological severity classification. This demonstrates that the spatial distribution of inflammatory cells holds clinical value equivalent to cell density in reflecting tissue structural alterations (30). Therefore, these AI classifiers can accurately diagnose EoE even on image patches with few eosinophils, achieving high accuracy (e.g., 85%-99%), sensitivity, and specificity (29).

The pathogenesis of eosinophilic esophagitis (EoE) is complex. Besides eosinophils, other immune cells (such as mast cells) may also be involved. Recent studies have begun to use AI to analyze other key immune cells. A study by Zimmerman et al. developed the Mast Cell-AI (MC-AI) tool for identifying, counting, and characterizing mast cells in EoE biopsy tissues (32). The MC-AI tool revealed that in active EoE, the density of intraepithelial mast cells increases, while that in the lamina propria decreases. Crucially, the density of mast cells in the epithelium and papillary regions is significantly correlated with the degree of eosinophilic infiltration, basal cell hyperplasia, and lamina propria fibrosis (32). In addition, Wu et al. investigated biomarkers for diagnosing EoE by integrating bioinformatics and machine learning analyses. The results indicated that CXC chemokine receptor 2 (CXCR2) is an independent diagnostic biomarker for pediatric eosinophilic esophagitis and is associated with immune cell infiltration, suggesting that regulatory T cells and neutrophils may play important roles in the pathogenesis of pediatric EoE (33). The lack of metrics like diagnostic time and subtle feature detection in these studies underscores the need for a comprehensive evaluation framework to fully assess AI’s endoscopic utility (**Table 1**).

## 4 AI for prediction, subtyping, and precision medicine in EoE

### 4.1 Early diagnosis of EoE and prediction of disease progression

The diagnosis of EoE relies on invasive endoscopic biopsy and histological evaluation, which may lead to diagnostic delays. Wang et al. developed a machine learning model incorporating patient demographics, hospital attributes and comorbidities to help clinicians screen high-risk EoE patients early and determine the need for biopsy. Validated to show good performance (34). Besides, a multicenter study collected patients’ demographic information, clinical symptoms, allergy history, comorbidities, and endoscopic findings, and developed and validated such prediction models using machine learning algorithms. The results showed that the model trained solely on clinical data exhibited good predictive performance in the external validation cohort (AUC 0.90, sensitivity 0.90, specificity 0.75). Moreover, the model combining clinical and endoscopic data performed better (AUC 0.94, sensitivity 0.94, specificity 0.68) (35) (20). This predictive model holds promise as an instant diagnostic tool. By integrating clinical and endoscopic examination data, it can forecast EoE risk prior to histology results, thereby assisting physicians in early risk stratification.

### 4.2 Application of AI in transcriptomics and molecular profiling analysis

EoE is a heterogeneous disease. AI-based ML algorithms, such as clustering analysis and predictive algorithms, are being employed to identify these endophenotypes from complex molecular data and construct predictive models (17). A landmark study analyzed esophageal mRNA transcriptomes using machine learning. Based on weighted analysis of genes such as eotaxin and periostin, the study developed a diagnostic probability score, p(EoE). A p(EoE) score of ≥25 identifies EoE with high accuracy (sensitivity 90.9%, specificity 93.2%). Additionally, this score changes with treatment, making it a potential tool for monitoring disease activity (9, 36). Furthermore, the study developed an IGHE score based on local IgE germline gene transcripts. This score can identify a subset of patients with highly allergic inflammation, who may be ideal candidates for IgE-blocking therapies (e.g., omalizumab) (36). Sallis et al. developed a diagnostic probability score (FI Score) using ML. This model enables early identification of food impaction risk in pediatric EoE patients by recognizing their individual molecular inflammatory signatures (37). Another study employed machine learning to comprehensively analyze histological, endoscopic, and molecular data, identifying three potential internal molecular phenotypes of EoE. The EoEe1 phenotype is characterized by mild inflammation and exhibits good response to steroid therapy. The EoEe2 phenotype, marked by prominent type 2 inflammation and predominantly childhood onset, shows poor response to steroids but may benefit from T2 pathway-targeted biologics (e.g., dupilumab) (38). These diagnostic tools established AI’s central role in EoE precision medicine by enabling a comprehensive “diagnosis-subtyping-prognosis” workflow. This integrated framework allows molecular profiling to directly guide clinical decisions.

### 4.3 Predicting therapeutic response: toward individualized EoE management

Currently, multiple studies have demonstrated the potential of AI in optimizing the diagnosis and treatment of esophageal reflux disease (EoE). For instance, by analyzing molecular signatures (such as microRNAs) in esophageal biopsy samples and combining this with machine learning modeling, it is possible to effectively distinguish responders from non-responders to proton pump inhibitor (PPI) therapy (7). Researchers also attempted to correlate AI-generated histological statistics (such as data based on the U-Net model) with therapeutic phenotypes (such as steroid responsiveness and dietary exclusion responsiveness) (29). Additionally, AI-based models can identify EoE patients likely to benefit from biologic therapy by analyzing transcriptomic and molecular biology data (17, 36). In terms of treatment strategy optimization, computer simulation models have been developed to identify the most effective dietary exclusion protocols, serving as an early example of using computational models to guide treatment (39). Additionally, Halder et al. developed a hybrid framework integrating fluid dynamics with machine learning, based on mechanical parameters such as esophageal wall stiffness and muscle contraction patterns. This framework has demonstrated clinical utility in evaluating treatment efficacy and monitoring patients’ post-treatment conditions (40) (**Table 1**). These studies established an AI-assisted framework that enhanced EoE treatment precision and patient tolerance through a “predict-monitor-optimize” workflow, providing a new paradigm for personalized care.

## 5 Common challenges in clinical implementation

First, the “black box” nature of deep learning models makes their decision-making processes opaque, hindering clinical trust and adoption (41, 42). While high accuracy is important, it is insufficient without an understanding of how the model arrives at a diagnosis. Therefore, emphasizing the need for model explainability through techniques like Explainable AI (XAI) is paramount. XAI technologies—such as Grad-CAM, which generates heatmaps—are particularly crucial when AI identifies novel biomarkers, as they reveal the basis for decision-making and are key to gaining clinicians’ trust (9, 30, 43). Secondly, existing studies are predominantly based on retrospective, single-center data, leaving a validation gap. Future studies should evaluate models not only on standard metrics but also on real-world performance across diverse hospitals, populations, and equipment. Comprehensive validation is currently the main barrier to clinical use. The model requires validation of its efficacy in an independent, multicenter prospective cohort (17). Additionally, the model exhibits limited versatility across different devices, populations, and environments, and faces complex and lengthy regulatory approval processes for medical devices (9, 44). Finally, at the data level, challenges related to privacy security and algorithmic fairness must be addressed. This requires employing techniques such as data anonymization and federated learning to safeguard data security, while ensuring the representativeness of training data to prevent exacerbating health inequalities (41, 45). Furthermore, the attribution of responsibility for AI errors and the establishment of clinical oversight mechanisms are also urgently needed (41).

## 6 Discussion and conclusions

This article reviews the remarkable success of AI in objective, image-based tasks for EoE diagnosis, with performance comparable to or even surpassing that of non-experts in many aspects (9). However, the current evidence requires critical appraisal. Most reviewed studies are retrospective, rely on single-institution data, and lack rigorous external validation across diverse populations and equipment. These limitations increase overfitting risks and hinder the direct translation of reported accuracy into clinical practice. Consequently, existing models should be regarded as proof-of-concept rather than clinically deployable tools. More importantly, the application of AI is shifting from automating existing tasks to discovering novel biomarkers and internal molecular phenotypes of patients, which represents a solid step toward truly precision medicine (36). Future research must prioritize addressing several key unmet needs in the current field: First, there is a severe lack of AI research on children and adolescents with EoE (17). Second, regarding the prediction of therapeutic response, developing models that can reliably predict patients’ responses to specific therapies remains one of the highest-priority tasks. Finally, using AI to develop or enhance non-invasive monitoring tools to replace repetitive invasive endoscopic examinations is the ultimate goal of current AI research (16, 43). The future of AI in the field of EoE lies not in isolated tools, but in an integrated, multimodal platform capable of integrating multidimensional data such as clinical, endoscopic, histological, molecular, and patient-reported outcomes (46).

In summary, despite ongoing significant challenges, AI technology is poised to transform EoE management from a reactive, “one-size-fits-all” model to a proactive, personalized, and data-driven science. Through earlier and more accurate diagnosis, more refined disease subtyping, and more predictive treatment selection, AI holds the promise of ultimately improving the quality of life for patients with this chronic condition.
